# Association of Fibroblast Growth Factor 23 With Ischemic Stroke and Its Subtypes: A Mendelian Randomization Study

**DOI:** 10.3389/fgene.2020.608517

**Published:** 2020-12-23

**Authors:** Kai Zheng, Lingmin Lin, Pan Cui, Tao Liu, Lin Chen, Chunsheng Yang, Wei Jiang

**Affiliations:** ^1^Department of Neurology, Tianjin Neurological Institute, Tianjin Medical University General Hospital, Tianjin, China; ^2^School of Brain Science and Brain Medicine, Zhejiang University, Hangzhou, China; ^3^Department of Neurology, The Affiliated Hospital of Qingdao University, Qingdao, China; ^4^Department of Physical Medicine and Rehabilitation, Tianjin Medical University General Hospital, Tianjin, China

**Keywords:** ischemic stroke, large-artery atherosclerotic stroke, Mendelian randomization, fibroblast growth factor 23, MEGASTROKE consortium, vitamin D regulation

## Abstract

Fibroblast growth factor 23 (FGF23), which is involved in the regulation of vitamin D, is an emerging independent risk factor for cardiovascular diseases. Previous studies have demonstrated a positive association between FGF23 and stroke. In this study, we aimed to assess the association of FGF23 with ischemic stroke and its subtypes by applying a Mendelian randomization (MR) framework. Five genetic variants obtained from a genome-wide association study involving 16,624 European subjects were used as valid instruments of circulating FGF23 levels. MR was applied to infer the causality of FGF23 levels and the risk of ischemic stroke using data from the MEGASTROKE consortium. Subsequently, several MR analyses, including inverse-variance weighted meta-analysis, MR-Egger, weighted median estimate (WME), MR Pleiotropy Residual Sum and Outlier were performed. The heterogeneity test analysis, including Cochran’s Q, *I*^2^ test and leave-one-out analysis were also applied. Furthermore, potential horizontal/vertical pleiotropy was assessed. Lastly, the power of MR analysis was tested. Three validated variants were found to be associated with circulating FGF23 levels and were used for further investigation. We found that high expression level of FGF23 was not associated with any ischemic stroke. However, a causal association between genetically predicted FGF23 levels and the risk of large-artery atherosclerotic stroke (LAS) was significant, with an odds ratio of 1.74 (95% confidence interval = 1.08–2.81) per standard deviation increase in circulating FGF23 levels. Our findings provide support for the causal association between FGF23 and LAS, and therefore, offer potential therapeutic targets for LAS. The specific roles of FGF23 in LAS and associated molecules require further investigation.

## Introduction

Stroke is one of the major causes of death and long-term disability worldwide (GBD 2016 Stroke Collaborators, 2019). Approximately 70% of strokes are ischemic stroke (IS), which is usually caused by the occlusion of the middle cerebral artery (The GBD 2016 Lifetime Risk of Stroke Collaborators et al., 2018). The increasing global burden and limited therapy options for stroke have led to urgent demands for more effective preventive and therapeutic measures (Avan et al., 2019).

Fibroblast growth factor 23 (FGF23), a bone-derived hormone, plays an important role in the regulation of calcium, phosphate, and active vitamin D levels (Vervloet, 2019). Recently, increasing evidence has indicated a strong relationship between FGF23 and cardiovascular diseases (Panwar et al., 2018). Several studies have demonstrated that an increased circulating FGF23 level was correlated with a higher risk (Wright et al., 2014) and a poorer outcome (Seiler et al., 2010) for stroke. Other studies have indicated that plasma FGF23 was associated with carotid atherosclerosis in patients who suffered from stroke as well as in the normal population (Shah et al., 2015; Yan et al., 2017; Chang et al., 2020). Meanwhile, higher FGF23 level also correlated with increased instability of carotid plaques (Biscetti et al., 2015). However, a case–cohort study indicated that there was a graded association of FGF23 with the risk of cardioembolic stroke, but there was no significant association between FGF23 and other IS subtypes or with hemorrhagic strokes in community-dwelling adults (Panwar et al., 2015). In addition, a Multi-Ethnic Study of Atherosclerosis (MESA) showed that FGF-23 was not associated with carotid intima-media thickness or stroke (Kestenbaum et al., 2014). Until now, it is unclear whether FGF23 levels are causally associated with risk of IS. Therefore, in this study, we aimed to investigate the possible causal relationships of FGF23 with IS and its subtypes and the potential research value of FGF23.

Recently, with the development of whole-genome association studies (GWAS), an increasing number of single-nucleotide polymorphisms (SNPs) related to human diseases have been identified (Pei et al., 2019; Liu et al., 2020a,b). Meanwhile, Mendelian randomization (MR) has been widely used for causal inference (Davies et al., 2018; Larsson et al., 2019). Since genetic variants such as SNPs are randomly allocated during conception and the genotypes are determined in the zygote stage, the MR framework can detect causality by minimizing the impacts of confounders and reverse causality (Davies et al., 2018). In this study, an MR design was used to investigate the association of circulating FGF23 levels with IS and its subtypes.

## Materials and Methods

### Study Design

**FIGURE 1:**
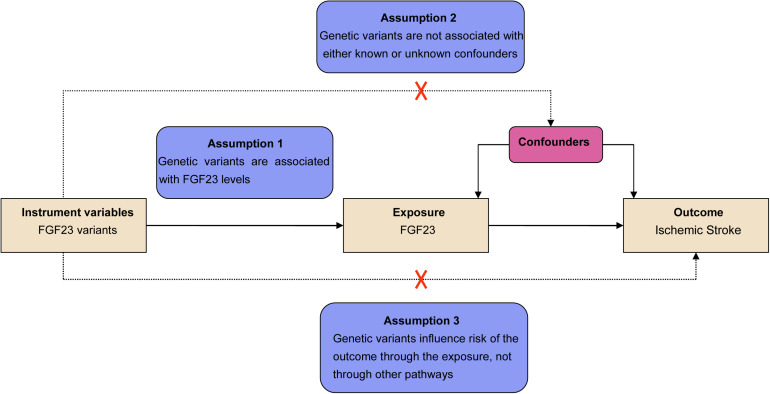
Assumptions for the Mendelian randomization (MR) and study design. The MR was based on three principal assumptions, including: (1) the genetic variants selected to be instrumental variables should be correlated with the exposure [fibroblast growth factor 23 (FGF23) levels]; (2) the genetic variants should be unrelated to confounding factors; (3) genetic variants must influence the risks of the outcome [ischemic stroke (IS)] only through the exposure (FGF23 levels).

MR was performed based on three primary assumptions as described previously (Yang et al., 2019; He et al., 2020). The first assumption was that the SNPs identified to be the instrumental variables (IVs) should be significantly related to the exposure (FGF23) (Figure 1). The second assumption was that genetic variants should be unrelated to the confounding factors of an outcome (IS) (Liu et al., 2018). The third assumption was that the genetic variants must only affect the risk of the disease (IS) through the exposure (FGF23) but not via other routes. Meanwhile, both the second and third assumptions were identified to be independent of pleiotropic effects. As the large-scale datasets from the published genome-wide meta-analysis were publicly available, no additional ethical approval was required.

### Selection of SNPs and Validation

The circulating FGF23-associated variants were collected from a meta-analysis comprising 16,624 individuals of European-descent after excluding those whose estimated glomerular filtration rate was less than 30 mL/min/1.73 m^2^ (Robinson-Cohen et al., 2018). The selected genetic instruments from the GWAS of FGF23 were composed of top five significant (*P* < 5 × 10^–8^) SNPs near *CYP24A1*, *ABO*, *RGS14*, *LINC01506*, and *LINC01229* genes, and were located in five genomic regions, accounting for approximately 3% of FGF23 variation. Detailed information is provided in Supplementary Table 1. The strength of the IVs was evaluated using the mean F-statistic, defined as the ratio of the mean square of effect size to the mean square of standard error for each genetic instrument (Bowden et al., 2016b). The rule of thumb threshold of F value is greater than 10 to avoid potential bias from weak instruments (Burgess and Thompson, 2011). The F statistics for each of the five instruments was greater than 10 (Supplementary Table 1). Subsequently, we verified the independence among these SNPs by linkage disequilibrium (*R*^2^ < 0.1) through the 1000 Genomes Phase 3 (European) reference panel.

### Data Sources

The summary-level data for IS and its subtypes were obtained from the MEGASTROKE consortium. Any ischemic stroke (AIS) group (*n* = 34,217), regardless of the subtype of European ancestry, was selected and compared with 406,111 control subjects. The three main subtypes of IS were acquired mainly on the basis of the Trial of ORG 10172 in Acute Stroke Treatment criteria, including LAS (*n* = 4,373), cardioembolic stroke (CES; *n* = 7,193), and small-vessel stroke (SVS; *n* = 5,386) (Malik et al., 2018). As all the five genetic instruments associated with FGF23 levels were available in the MEGASTROKE consortium, no proxy variant was needed. The MEGASTROKE-matched data are shown in Supplementary Table 2.

### Statistical Analysis

The principal analyses assessing the causal associations of FGF23 with IS and its subtypes were performed using the inverse-variance-weighted (IVW) method (Davies et al., 2018). For each of the five SNPs, we computed an Wald’s ratio estimates by dividing the beta-coefficients (log odds ratio) for the SNP–stroke association by the beta coefficient for the SNP–FGF23 association. Moreover, to improve the reliability of causal effect estimates, we also carried out the MR Pleiotropy Residual Sum and Outlier (MR-PRESSO) test (Verbanck et al., 2018).

To further evaluate the impact of potential pleiotropy on causal estimates, we performed sensitivity analyses using several other methods. First, we used the MR-Egger regression to assess the presence of directional pleiotropy (Bowden et al., 2015). A statistically significant intercept term from the MR-Egger regression suggests the possibility that genetic variants may not affect the outcome via the exposure of interest. We also conducted the weighted median estimate (WME) (Bowden et al., 2016a), which provides an effective estimate of causality when at least 50% of genetic IVs is valid. Furthermore, to evaluate the potential heterogeneity due to pleiotropy or other causes, we conducted the Cochran’s *Q*-test (together with the *I*^2^ statistic), as reported in a previous study (Liu et al., 2013). In addition, we selected the leave-one-out sensitivity method to sequentially remove each SNP from the MR analysis and assess the impact of single-gene variants on the causal estimates (He et al., 2020). Moreover, vertical pleiotropy was assessed using the Steiger test to verify the causal direction between FGF23 and stroke (Hemani et al., 2017).

Lastly, we excluded those SNPs associated with potential confounders (Bonferroni correction, *P* < 0.05/5 SNPs) by using PhenoScanner V2 in March 2020 (Staley et al., 2016; Kamat et al., 2019), and repeated the MR analysis using the IVW, MR-Egger regression, and weighted-median estimate. To correct for potential pleiotropic bias, we performed multivariable MR following Sanderson’s method (Sanderson et al., 2020). We also calculated the power of MR estimates using the mRnd platform^1^ and the effect size based on a 5% type 1 error rate and enough power (>80%). Statistical analyses were performed using Mendelian Randomization (version 0.4.1) (Yavorska and Burgess, 2017) and TwoSampleMR (version 0.5.1) (Hemani, 2019) on R 3.6.2 (The R Foundation for Statistical Computing, Vienna, Austria). All statistical tests were two-sided and the statistical significance was set at the level of *P* < 0.05.

## Results

### Primary MR Analysis of the Association of FGF23 With IS and Its Subtypes

As listed in Supplementary Table 1, five SNPs were used as the IVs for FGF23 levels. We identified significant association of high FGF23 levels with increased LAS risk (OR = 1.94, 95% CI 1.35–2.27; *p* = 3.04E−04) but not with the other IS subtypes or AIS using the IVW method (Supplementary Table 4). However, a potential heterogeneity was identified using the Cochran’s Q test and *I*^2^ for causal estimates of five SNPs in the conventional IVW model for AIS (14.34, *p* = 0.0063, *I*^2^ = 72.10%), LAS (14.12, *p* = 0.0069, *I*^2^ = 71.70%), and CES (16.79, *p* = 0.0021, *I*^2^ = 76.20%) (Supplementary Table 4), suggesting the possibility that the obtained effect estimates of these associations from the IVW method may be biased by outlier SNPs.

### Sensitivity Analysis of the Association of FGF23 With IS and Its Subtypes

To assess the robustness of the causal effect of FGF23 on IS and its subtypes, we performed several sensitivity analyses as follows. First, WME suggested significant association between FGF23 levels and LAS risk with an odds ratio of 1.75 (95% CI 1.06–2.90; *p* = 0.029), but not with the other IS subtypes or AIS (Supplementary Table 4). Second, the intercept term from MR-Egger analysis revealed no evidence of directional pleiotropy in the analysis of LAS (*p* = 0.81), SVS (*p* = 0.97), CES (*p* = 0.22), or AIS (*p* = 0.26). However, MR-PRESSO test identified horizontal pleiotropic outliers in AIS (*p* = 0.0066), LAS (*p* = 0.0134), and CES (*p* = 0.0034). The leave-one-out permutation analysis further indicated that the direction and precision of the genetics estimates between increased FGF23 levels and risk of IS and its subtypes changed largely with the deletion of *rs2769071* (Supplementary Table 7).

We next searched the PhenoScanner V2 database (Kamat et al., 2019) for possible pleiotropic associations of individual SNPs with risk factors for IS. Among the FGF23-associated SNPs, associations were observed for the *rs2769071* variant with low-density lipoprotein (*P* = 3.06E−10), total cholesterol (*P* = 7.48E−13), diastolic blood pressure (*P* = 2.80E−10), and type 2 diabetes (*P* = 2.30E−05). The *rs11741640* variant was significantly related to self-reported hypertension (*P* = 2.22E−04) and alcohol intake frequency (*P* = 3.74E−03). Detailed information is provided in Supplementary Table 3.

**FIGURE 2:**
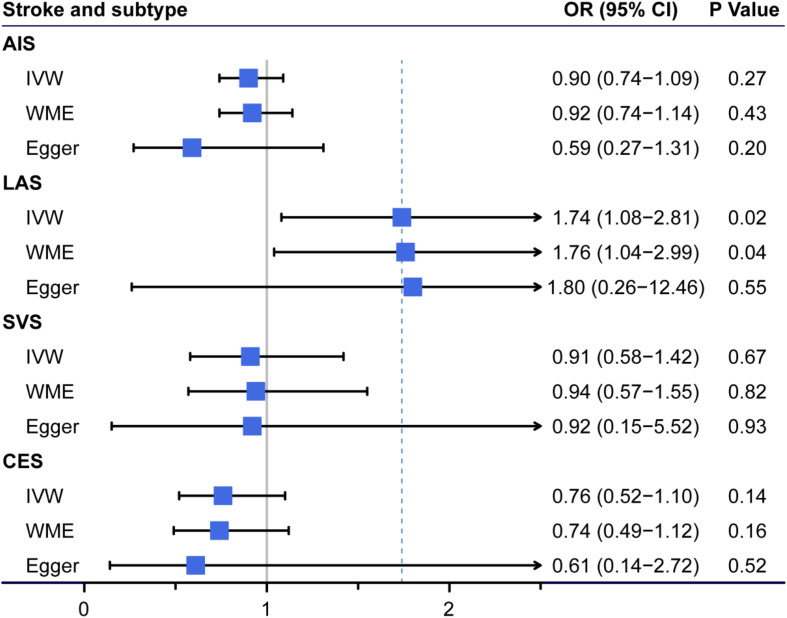
Association of genetically predicted circulating fibroblast growth factor 23 (FGF23) levels with ischemic stroke (IS) and other stroke subtypes. The odds ratio (OR) represented at the center of each box was the risk of genetically predicted one standard deviation increase in FGF23 levels. AIS, any ischemic stroke; LAS, large-artery atherosclerotic stroke; SVS, small-vessel stroke; CES, cardioembolic stroke; CI, confidence interval; IVW, Mendelian randomization (MR) inverse-variance weighted method; WME, weighted median estimate; Egger, the MR-Egger method.

In total, we excluded two SNPs (*rs2769071* near the *ABO* gene and *rs11741640* near the *R*GS14 gene) that were potentially associated with at least one secondary phenotype and repeated the MR analyses. Based on the remaining three effective SNPs, FGF23 levels were significantly associated with LAS but not with the other IS subtypes or AIS (Figure 2). In the standard MR analysis-IVW method, the odds ratios per standard deviation of the genetically predicted increase in FGF23 levels was 1.74 (95% CI 1.08–2.81; *p* = 0.023) for LAS. Importantly, the results obtained for LAS were similar in the WME analysis (OR = 1.76, 95% CI = 1.04–2.99; *p* = 0.036), while the Egger estimate was less precise despite having the same direction and a similar size (OR = 1.80, 95% CI = 0.26–12.46; *P* = 0.549). The single variant causal ratio and results of all three variants for the association of FGF23 and LAS are shown in Supplementary Table 6. No heterogeneity among these three instruments was found using Cochran’s Q analysis (*Q* = 0.02, *P* = 0.992, *I*^2^ = 0.00%) in LAS (Supplementary Table 5). The leave-one-out sensitivity analysis also showed the same direction and estimates between the increased FGF23 levels and the risk of LAS, although the deletion of IV *rs17216707* near *CYP24A1* gene was not statistically significant (Supplementary Table 8). No directional pleiotropy in LAS was found according to the Egger intercept test (–0.003, 95% CI = –0.150 to 0.145; *P* = 0.970). Considered the potential effects of obesity and smoking-the two most important confounders for both heart disease and circulating metabolites, we then applied the multivariable MR analysis. The BMI or smoke adjusted data by three validated instruments also verifies our results (Supplementary Table 9).

Besides, the direction of causality inferred by the Steiger test showed that the SNPs–FGF23 association (*r*^2^ = 1.04E−02) was more significantly correlated (*p*_Steiger_ = 3.20 × 10^–14^) than the SNPs-LAS association (*r*^2^ = 1.26E−05), suggesting that higher FGF23 levels leads to the increased risk of LAS, consistent with expectation. We had enough power (>80%) to detect 1.59 OR of LAS risk per SD increased log FGF23 levels (cases *n* = 4,373; non-cases *n* = 406,111); and the power of causal estimate for FGF23 to LAS here was 94%.

## Discussion

Until now, it has remained unclear whether the circulating FGF23 levels is genetically associated with risk of IS. In this study, we found a potential association of genetically predicted high levels of FGF23 and the increased risk of LAS. The risk of LAS increased by 74% with a 23 pg/mL per SD increase in circulating FGF23 levels. This effect size was similar to previously reported sizes of low-density lipoprotein cholesterol (OR = 1.28, 95% CI = 1.07–1.53) (Hindy et al., 2018), fasting blood glucose (OR = 1.42, 95% CI = 1.08–1.85) (Larsson et al., 2017), systolic blood pressure (OR = 1.56, 95% CI = 1.37–1.78) (Parish et al., 2019), and waist-to-hip ratio (OR = 1.75, 95% CI = 1.44–2.13) (Marini et al., 2020).

Our results are consistent with those of previous epidemiological studies (Seiler et al., 2010; Shah et al., 2015; Yan et al., 2017; Chang et al., 2020). In patients with acute IS, the plasma FGF23 concentration was positively correlated with the presence and burden of intracranial carotid atherosclerosis (Chang et al., 2020). FGF23 seems to be mainly involved in vessel calcification, vascular stiffness, and inflammation (Mirza et al., 2009; Kim et al., 2011; Libby et al., 2019; Vervloet, 2019). In mice, excessive plasma FGF23 directly stimulates the production of inflammatory factors such as interleukin-6 (Singh et al., 2016). Meanwhile, inflammatory factors in turn promote the production of FGF23 and exacerbate LAS progression (Feger et al., 2017; Durlacher-Betzer et al., 2018; Egli-Spichtig et al., 2019; McKnight et al., 2020). Our analysis implied that reducing FGF23 levels may be a potential therapeutic strategy for IS, especially for LAS. However, the potential mechanisms that correlate FGF23 with LAS still require further investigations.

Considering the role of FGF23 in regulation of vitamin D levels, some previous studies argued that the pathophysiological effects of FGF23 were partially through decreasing the level of active vitamin D. FGF23 inhibits the functions of vitamin D by promoting its degradation via 24-hydroxylase encoded by the *CYP24A1* gene and inhibiting its production by 1α-hydroxylase encoded by the *CYP27B1* gene (Vervloet, 2019). The physiological roles of vitamin D, including anti-inflammation and inhibition of artery calcification, are contrary to the effects of FGF23 (Han et al., 2016; Wang et al., 2018). In addition, vitamin D receptor activation enables the recovery of αKlotho, an anti-aging protein, while this recovery is inhibited in an inflammatory environment (Lim et al., 2012). FGF23 induces vessel damage and inflammation through an αKlotho-independent pathway when αKlotho is insufficient (Komaba and Fukagawa, 2012; Navarro-González et al., 2014; Krick et al., 2018). The aforementioned studies collectively suggest that proper calcitriol supplements might reduce the risk of LAS in people or those with intracranial atherosclerosis. The effects of calcitriol supplements involved in the process of vasomotion and immune modulation have been reported by several studies (Chitalia et al., 2014; Ojeda López et al., 2018). In this study, the validated genetic variant (*rs17216707*) near the *CYP24A1* gene showed a strong association with LAS (Supplementary Table 6), which supports the critical role of active vitamin D in the regulation of FGF23 level and the risk of LAS.

To our knowledge, this is the first MR study to clarify the genetic causalities between FGF23 levels and IS with MR methods. Considering the ethical care of patients and the high cost of randomized controlled trials, the MR framework is effective in the discovery of potential targets of intervention and can indicate potential therapeutic strategies. In addition, our findings in this study were especially prospective, as analyzed data were extracted from the database with the largest number of participants currently known.

However, this study also has several limitations. The different methods for FGF23 measurement could have potentially caused bias in the results. The FGF23 levels were detected in two forms: intact and C-terminal FGF23 (Robinson-Cohen et al., 2018). In patients with chronic kidney diseases, the production of FGF23 (intact FGF23) was separated from its cleaved form (C-terminal FGF23) (Edmonston and Wolf, 2020). Meanwhile, the FGF23-associated GWAS data were obtained from individuals whose estimated glomerular filtration rate was above 30 mL/min/1.73 m^2^. In addition, log-transformed FGF23 levels, applied in each cohort and the following meta-analysis, could reflect the relative change in circulating FGF23 levels.

In our study, only three SNPs accounting for 1.13% of the total variation in FGF23 levels were identified as genetic instruments, causing a possible limitation in the results. Thus, additional influential loci are necessary as IVs in the future if new GWAS data are available. As this limited number of IVs restricted the application of PRESSO, the sensitive analysis of potential horizontal pleiotropy could not be performed completely. However, similar results were obtained from WME and IVW estimate, while no signs of heterogeneity (Cochran’s Q test) and directional pleiotropy (MR-Egger intercept analysis) were discovered. Therefore, the above results indicated that confounders are unlikely to explain the observed associations.

Population stratification also potentially restricted the accuracy of this study. The MR inference depended on three instrumental assumptions that rely on the same genetic backgrounds in the exposure and outcome data. In this study, we used European-descent genotypes to assess the association between FGF23 levels and IS. This result may be altered in different populations due to different genetic backgrounds, such as linkage disequilibrium. Moreover, the MR framework was not able to infer the association during specific periods of the life cycle or conditions. Thus, further animal experiments and possible intervention trials are needed.

In summary, our results provide support for a suggestive causal association between higher circulating FGF23 levels and an increased risk of LAS. Our findings may offer new therapeutic targets for LAS. Further studies are necessary to investigate whether genetic variants at or near the *CYP24A1* gene influence the risk of LAS through downstream effects or pathways related to vitamin D.

## Data Availability Statement

The datasets presented in this study can be found in online repositories. The names of the repository/repositories and accession number(s) can be found in the article/Supplementary Material.

## Author Contributions

LL, CY, KZ, and PC contributed to the conception and design of the study. LL collected data and performed the MR framework. KZ and LL drafted the manuscript. KZ, LL, PC, TL, LC, CY, and WJ participated in the analysis of the results and provided critical review. All authors approved the submitted article.

## Conflict of Interest

The authors declare that the research was conducted in the absence of any commercial or financial relationships that could be construed as a potential conflict of interest.

## Footnotes

1 https://shiny.cnsgenomics.com/mRnd/

## Abbreviations

AIS: any ischemic stroke
CES: cardioembolic stroke
CI: confidence interval
FGF23: fibroblast growth factor 23
GWAS: whole-genome association studies
IS: ischemic stroke
IVW: inverse-variance weighted
LAS: large-artery atherosclerotic stroke
MR: Mendelian randomization
MR-PRESSO: MR pleiotropy residual sum and outlier
OR: odds ratio
SNPs: single-nucleotide polymorphisms
SVS: small-vessel stroke
WME: weighted median estimate.
